# What is needed to improve quality of implant removal services in Nigeria? results of a landscape assessment

**DOI:** 10.3389/fgwh.2023.1082969

**Published:** 2023-03-22

**Authors:** Oniyire Adetiloye, Abubakar Danladi, Rachel Haws, Charity Anoke, Bartholomew Odio, Emmanuel Ugwa, Agnes Nganje, Joseph Enne, Kayode Afolabi, Owodunni Adebola, Justus Eze, Megan Christofield

**Affiliations:** ^1^ Jhpiego, Abuja, Nigeria; ^2^ Department of Obstetrics and Gynaecology, Federal Medical Centre Gusau, Gusau, Nigeria; ^3^ Department of International Health, Johns Hopkins Bloomberg School of Public Health, Baltimore, MD, United States; ^4^ Department of Obstetrics and Gynaecology, Federal Medical Centre Bernin, Kudu, Nigeria; ^5^ Reproductive Health, Federal Ministry of Health, Abuja, Nigeria; ^6^ Department of Obstetrics and Gynaecology, Federal Medical Centre Abakaliki, Abakaliki, Nigeria; ^7^ Jhpiego, Baltimore, MD, United States

**Keywords:** long-acting reversible contraceptive, difficult removal, non-palpable, quality of care, quality improvement

## Abstract

**Introduction:**

Stunning recent increases in subdermal contraceptive implant use, especially in sub-Saharan Africa, necessitate availability of quality implant removal services. In Nigeria, service delivery capacity and coverage for removal are lacking, despite strong government commitment and rapid uptake; there is a dearth of knowledge about barriers to quality implant removals in Nigeria.

**Methods:**

To determine access to and quality of contraceptive implant removal services, a landscape assessment was conducted in two states in Nigeria, focusing on four conditions for quality delineated in the Global Implant Removals Task Force framework. This mixed-methods approach integrated results from a desk review, a survey of health facilities and family planning managers, review of implant service statistics, and key informant interviews with providers and diverse stakeholders.

**Results:**

Seventy percent of providers (*N* = 21 of 30) had experienced problems performing implant removal, usually due to deeply inserted implants and equipment shortages. Providers had low confidence in performing removal and poor knowledge of implant removal steps. No facilities assessed had comprehensive equipment required for implant removal. Few facilities maintained systems or referral pathways to support difficult removals; difficult removals are absent from training manuals, and no formal trainings have been conducted. While most facilities collect data on removals, family planning dashboards do not capture it; few facilities use data for quality improvement.

**Conclusion:**

This study identified numerous challenges to quality implant removal, including poorly trained providers, inadequate supplies, underutilization of data on removals, and inability to manage difficult removals. As demand for implant removals skyrockets, providers need improved training in implant removal, appropriate job aids, supportive supervision, and effective procurement systems to ensure availability of supplies and equipment for removal. Tracking removals and reasons for removal in information systems and the Family Planning dashboard could sensitize providers to need for implant removals and improve data for decision-making in facilities and health systems.

## Introduction

Over the last decade, use of subdermal contraceptive implants has increased more than any other contraceptive method in global popularity and uptake, particularly in sub-Saharan Africa (1–3). The Implants Access Program, a public-private multi-country collaboration launched in 2013, decreased unit cost of implants for women in low- and middle-income countries; strengthened supply chain performance; trained providers in insertion and removal; and increased knowledge and awareness about long-acting reversible contraceptives (LARCs) at the community level (4). The Implants Access Program led to a 10-fold increase in procurement from 2010 to 2018, and has been credited with dramatically increased prevalence of contraceptive implant use in countries participating in the FP2020 initiative, including Nigeria (4). The modern contraceptive prevalence rate in Nigeria has increased from 11 percent in 2013 to 17.6 percent in 2018 (5, 6); an 8.4-fold increase in implant uptake accounts for much of this increase (7–9). Implants are now the most common contraceptive method in Nigeria among married women; both two-rod levonorgestrel (“Jadelle”) and one-rod etonogestrel (“Implanon”/“Implanon NXT”) implants are available (10). In 2017, the Nigerian government committed to offering FP services free of charge (including all consumables and consultation fees), and Nigeria's National Family Planning Blueprint 2020–2024 calls for increased access to implants and removal services, supported through increased numbers of trained providers, and mentorship and supervision to improve service provision (11). Since 2014, trained community health extension workers have been permitted to perform insertion and removal services; in remote areas, these workers may be the only available providers for implant services (12). Because private facilities are a major source of family planning services, the government is attempting to scale up private providers' access to free FP commodities, but thus far only a few private facilities have benefited from this initiative (13). In reality, exorbitant user fees are still charged in many public and private facilities, counter to established policy and guidelines, which limits access to implant services.

Currently available contraceptive implant products have a lifespan of three to five years, though users can discontinue use early if desired. As changing user preferences and expanded availability of LARCs lead more women to choose implants, a parallel need arises for services for timely removal, either because the implant is reaching the end of its lifespan or the user desires removal (3, 6, 14, 15). Countries like Nigeria that rapidly scaled up contraceptive implants in recent years have thus reached a critical period regarding provision of removal services. Access to quality implant removal services is a critical element of informed choice in implant use (3, 16). Provision of removal services is also integral to the success of countries' family planning (FP) program objectives because removal experiences influence satisfaction with and demand for implants (17).

However, mounting evidence indicates that women often lack access to high-quality implant removal services (18, 19). Generally, implants are easy to remove through a small opening in the skin. On rare occasions, implants are difficult to remove due to being non-palpable, having migrated, having been inserted incorrectly, or having become encased in fibrous tissue. These difficult removals may require referral to other providers and/or facilities with appropriate capacity and ultrasound or x-ray availability (20). Adequate planning, resource allocation and placement, and data to drive quality improvement are needed to ensure availability of these services, particularly for difficult removals (21).

Based on the volume of implants procured in Nigeria in recent years, an estimated 5.1 million removals will be needed before 2025 (22). However, emerging data indicate that service delivery capacity for implant removals has not kept pace with that for insertion in FP2020 countries, and numerous barriers hinder access to quality implant removals. Clinical challenges can result in failed removal services, supply chain issues can limit availability of needed consumables, and geographic challenges can adversely affect clients seeking removal (facilities offering removals may be farther away than those offering insertions (23). Several studies describe users seeking implant removal from providers but failing to obtain them when desired (3, 16, 24) Where removal services are readily available, studies have found that up to 20 percent of implant users request removals during the first year of use (25, 26). However, a review of service statistics in three sub-Saharan African countries by EngenderHealth reported 136,737 insertions and only 4,092 removals between January 2014 and June 2016 (27). Even acknowledging that data on removals is far from complete, Sergison et al. note that the three percent removal rate over that two-year study period suggests insufficient access to removal services (28).

**Figure 1:**
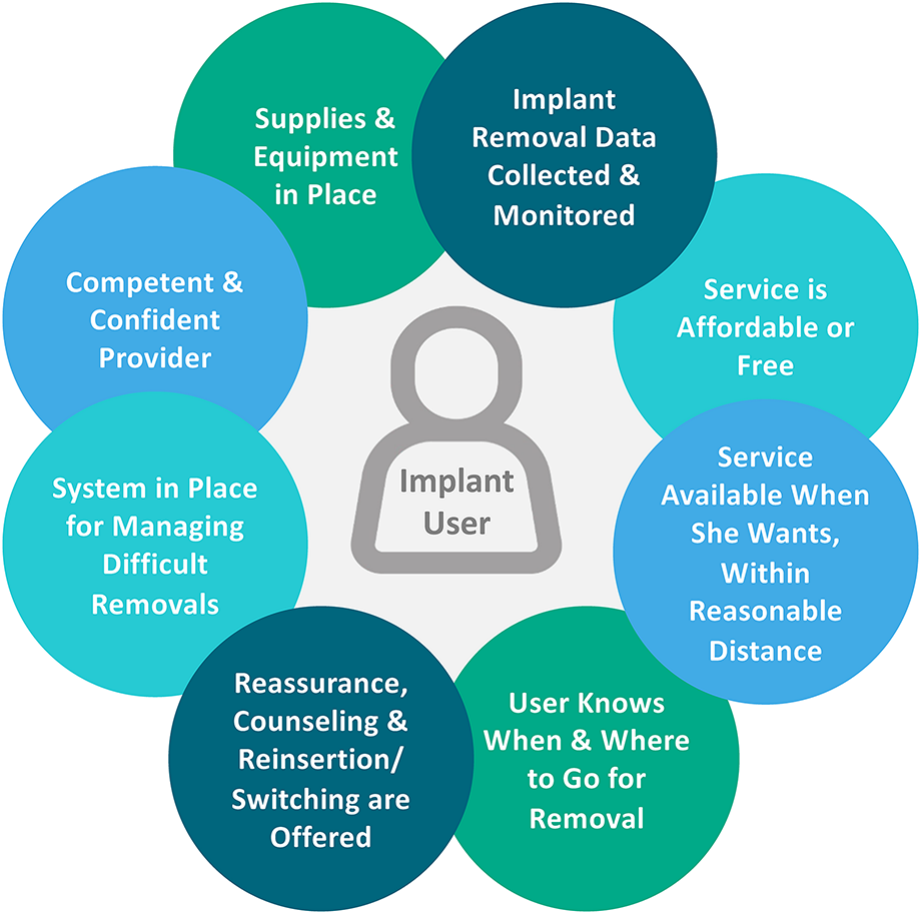
Client-centered conditions for ensuring access to quality implant removal (via Implant Removals Task Force, with permission).

Improving access to quality implant removal meets a fundamental client right to full, free, and informed choice both to use and to discontinue a selected contraceptive method (3, 28). To this end, the Global Implant Removal Task Force, initiated in 2015 as part of the Implants Access Program Operations Group, developed a framework of client-centered conditions that must be met to ensure the availability of quality implant removal services (Figure 1) (3). Four of the eight client-centered conditions that must be met for quality removals can be addressed through quality improvement efforts at the facility level (29–31): a competent and competent provider, supplies and equipment in place, a system in place for managing difficult removals, and Implant removal data collected and monitored. Quality of implant removal is provider dependent: training experience, clinical practice, and adequate client loads following training help maintain skills required for removal. Determining the capability of providers to offer quality service requires assessment of these factors (3). Referral protocols, availability of equipment and job aids, supportive supervision/mentoring, and other quality improvement activities support capacity building for quality implant removal services, particularly in cases of difficult removal. Last, systematic capture, synthesis, and analysis of data on removals is integral to developing action plans, scaling up access, and ensuring accountability in providing this essential service (21). However, few countries routinely track removals and reasons for removal.

Significant effort and investment in training providers has facilitated rapid uptake of implants in Nigeria, but little is known about access to and quality of implant removal services. Jhpiego—a non-profit health organization affiliated with the Johns Hopkins University—led a landscape assessment to determine access to and quality of contraceptive implant removal services in selected sites in Nigeria. This assessment was part of the Expanding Family Planning Choices project in Nigeria, a program funded by the Bill & Melinda Gates Foundation to identify service gaps and integrate a high-quality implant removal component into the country's overall implant strategy.

## Materials and methods

### Study design

The landscape assessment employed a cross-sectional mixed methods approach, aiming to capture information from a wide range of stakeholders at multiple levels. Assessment questions were developed after consultation with the project team and the Federal Ministry of Health (FMOH) Nigeria. Potential focus areas were ranked based on several criteria, including perceived need, potential magnitude of impact, and congruence with Jhpiego's technical focus. Based on modifiability at the facility level using the criteria above, four of the eight client-centered conditions in the Global Implant Removals Task Force framework were prioritized: (1) Competent and competent provider, (2) Supplies and equipment in place, (3) System in place for managing difficult removals, and (4) Implant removal data collected and monitored (Figure 1). A desk review of relevant documents was conducted to evaluate the policy and program environment relating to these prioritized client-centered conditions of quality implant removal. The desk review was then used to inform development of data collection tools for (1) a retrospective analysis of service statistics of implant services and related data at 12 study facilities for the 6 months prior to the survey; (2) a semi-structured survey of provider knowledge and confidence at study health facilities; (3) a semi-structured survey of facility managers at study facilities on facility readiness, and (4) qualitative key informant interviews (KIIs) with national and state-level stakeholders, health care workers, and implementing partners around the four prioritized client-centered conditions.

### Pre-assessment desk review

The desk review compiled and examined reports, policy documents, work plans, guidelines, training manuals, notes from stakeholder meetings, and other relevant literature and publications to evaluate the current context around the four prioritized client-centered conditions for implant removal. Data were extracted from these sources were reviewed for relevance to the four prioritized client-centered conditions using a standard data extraction template that captured information on a wide range of related topics and objectives of the assessment relevant to the client-centered conditions as well as broader aspects of implant service provision and family planning. Documents for the desk review were identified and obtained *via* online searches in Google Scholar and PubMed, as well as in response to requests the study team made to FMOH, state ministries of health, and implementing partners for relevant gray literature.

### Study setting

**Figure 2:**
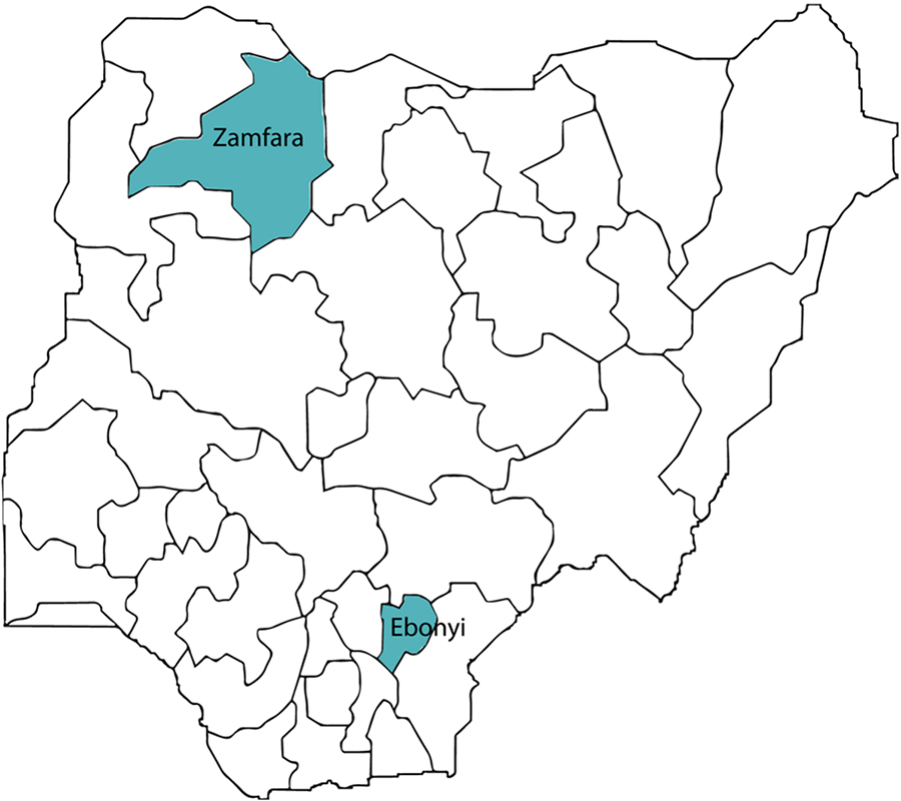
Map of Expanding Family Planning Choices project area.

The assessment took place in two states of Nigeria (Ebonyi and Zamfara states) (Figure 2). These states were purposively selected based on several inclusion criteria considering available resources, time constraints, and logistic requirements. States selected had prior and current presence and support of implementing partners to provide contraceptive implant services, and had robust implant service provision in place (defined as availability of trained service providers for implant services and consistent uptake of implant services). One state in the northwest (Zamfara) and one in the southeast (Ebonyi) were selected to maximize geographic diversity; both have low contraceptive prevalence rates (5.65 and 15.7 percent, respectively) and high fertility rates (6.8 and 5.3, respectively) (32). Zamfara has a longer history of implant service provision due to implementing partner presence (including the Accelerated Scale of Implant project), and has adopted a task-shifting policy, where community health extension workers have been trained to provide implant insertions. Almost half (48 percent) of facilities in Zamfara State had LARC-trained health workers, significantly higher than the national average of 18 percent (33). Ebonyi State has a large pool of active, trained implant providers, and high uptake of implants due to support provided by Jhpiego through the Maternal and Child Survival Program.

Data collection in Zamfara and Ebonyi states took place in October 2018 and January-February 2019, respectively. Key informant interviews with national-level stakeholders were conducted in December 2018.

### Sampling

Multi-stage sampling was used to select study health facilities and providers. Within each state, the six facilities with the highest number of trained providers and implant client load were selected. Each state's family planning and reproductive health coordinator, who had access to training records and a list of facilities providing implant services, guided selection of providers. In each state, two secondary hospitals, two primary health centers, one federally-owned tertiary hospital (to permit insights across different ownership structure) and one private health facility were selected, for a total of 12 health facilities. For key informant interviews with implementing partners, respondents were purposively selected based on active involvement with implant service provision at national level and in study states. For key informant interviews with national and state stakeholders, informants with direct responsibilities and involvement with implant service provision were selected. For key informant interviews with pre-service institutions, the head of the school of midwifery or a designee named by the head was selected.

### Study procedures

The study team included three experienced medical clinicians with expertise in public health research, who led the landscape assessment and conducted field activities; three additional team members with public health backgrounds contributed to design of the study, tool development, and data analysis and interpretation, with support of a statistician. The study protocol and assessment tools were developed in close collaboration with stakeholders, including members of the study team and FMOH. During field assessments, the study team first obtained permission to conduct the study from FMOH and state ministries of health, hospital management boards, and state primary health care agencies. The study team then sensitized the heads of selected health facilities and in-charges to the purpose of the research, asking hospital managers for assistance in establishing rapport with providers and explaining inclusion criteria. Participants for interviews were randomly selected from a list of trained implant service providers at each facility and engaged *via* a formal letter, after which a member of the study team scheduled the interview. No selected providers refused to participate. All data were stored in a password-protected web-based Research Electronic Data Capture (REDCap) application housed in a fully customized Jhpiego Cloud Server (34).

#### Retrospective analysis of service delivery statistics

To quantify implant insertions and removals, along with other indicators relating to quality of implant insertion and removal services, FP service delivery statistics were extracted for the 6 months prior to the assessment (October 2018–February 2019) from family planning service registers, service reports, and wall charts available in study facilities. Indicators extracted included numbers of implant insertions and removals, number of difficult removals (if recorded), as well as FP service delivery statistics summaries, monthly data review meeting summaries, and reporting to the state level ministry of health. The field investigator double-checked the main register to validate data obtained from summary registers and to synchronize information with notes and audio recordings before uploading to REDCap. No major discrepancies were encountered, but areas where additional clarity was needed were flagged for discussion in KIIs, and where there were discrepancies between summary registers and the main register, the main register data was used.

#### Semi-structured survey with providers on knowledge and confidence

To assess provider knowledge and confidence, a semi-structured survey was developed by the study team and conducted with implant providers at study facilities. Providers were deemed eligible if they were trained and actively providing implant insertion and removal services and consented to participate. A sampling frame of 32 providers was developed based on the assumption that there would be four eligible providers at tertiary and secondary hospitals, and two at primary health centers and private health facilities. Subsequent sample size calculations required interviews with 30 providers for a 5 percent level of error; the survey was administered to a total of 30 eligible providers across the 12 study facilities. At each facility, eligible providers (4–5 from each public facility and 1–2 from each private facility) were selected randomly from a list of trained implant providers at that facility prepared by the hospital manager, who also helped introduce the study to eligible participants. The questionnaire solicited information on trainings received, experience in providing implant insertion and removals (especially difficult removals), and support systems in place for removal services (e.g., mentoring, supportive supervision, job aids, and quality improvement committees). Providers were considered confident in implant service delivery if they rated their confidence as 4 or 5 on a scale of 1 to 5.

#### Semi-structured survey of facility managers on facility readiness

To assess facility readiness to provide quality implant insertion and removal services, a semi-structured data collection tool was developed by the study team based on a literature review and technical inputs from Jhpiego. The tool was used to interview the facility head of FP/Maternal and Child Health units at the total sample of all study facilities (*N* = 12). The instrument assessed the number of providers offering implant services and examined availability of equipment and supplies, health infrastructure, and job aids. This instrument also inquired about challenges with implant removal, referral practices, supportive supervision, and data management and reporting. The semi-structured questionnaire was programmed into Android devices used for data collection; built-in filters and controls minimized data entry and skip pattern errors in the questionnaire. The facility assessment also included a tour of the facility to observe equipment, supplies and registers. The interview and assessment took a minimum of 60 min and maximum of 120 min to complete.

#### Qualitative KIIs with stakeholders at state and national level

Respondents for these interviews were purposively selected based on their role in providing family planning services, particularly implants. Interviews were conducted to gain insight about the prioritized client-centered conditions for implant removal with national and state-level stakeholders (*N* = 5), including the reproductive health coordinator from each state and three respondents from the Ministry of Health. Additional KIIs (*N* = 16) with FP implementing partners responsible for technical and programmatic support for and coordination of implant provision—including Society for Family Health, United Nations Population Fund, Marie Stopes International, SHOP Plus, Pathfinder international, Nigeria Urban Reproductive Health Initiative, Jhpiego, and Planned Parenthood Federation of Nigeria—were conducted to explore capacity-building practices for implant removal, including targeted providers, training curriculum, duration of training, clinical training on actual clients, training on difficult implant removals, and post-training support activities. Last, key informant interviews using a semi-structured interview guide were conducted with principals/staff of pre-service institutions (*N* = 3, one in Zamfara and 2 in Ebonyi) to explore how specific facilities provide training on implant insertion and removal, as well as availability and adequacy of training materials (e.g., training curriculum and anatomical models). The sampling frame and size was intended to capture a diverse range of views from all major implant services supply-side stakeholders at all levels of the health system, and was conducted until saturation was reached.

Participants were identified in collaboration with FMOH and professional networks, and invited *via* formal letter. KIIs utilized a semi-structured questionnaire supported by probes when appropriate. Two members of the study team (DA and NA) conducted all surveys/interviews (primarily in person, especially for semi-structured interviews at health facilities; occasionally KIIs were conducted by phone based on participant preference); interviews were conducted in English and lasted an average of 45 min. One member of the study team documented responses in a data collection tool on an Android device; the other took notes and audio-recorded the interview, uploading data to REDCap. Interviews were held in an agreed-upon location providing auditory and visual privacy: either the key informant's office or a selected area of the facility. Information provided was only known to the study team members and respondents' supervisors were not informed of their responses. Each KII lasted approximately 45–60 min.

### Data analysis

The data manager conducted regular quality checks during data collection prior to data analysis. All data in REDCap were directly validated and cleaned to remove duplicates and invalid records and complete missing fields, and exported to Excel. After crosschecking for completeness, data were exported into SPSS version 25 for analysis (35). We used service delivery statistics to evaluate characteristics of health facilities providing implant insertions and removals in each state and overall by facility type, ownership, and monthly insertions and removals. Survey data from providers on confidence and knowledge were used to compute frequencies and percentages of health providers in each state reporting confidence in performing insertion and removal, providers who knew the correct steps in implant removal, and providers who had not had the opportunity to perform implant removal on a live client during training. Then, we used facility readiness survey data to tabulate the number and percentage of facilities in each state that were adequately addressing each of the four client-centered conditions. We also used facility readiness data to compute the number and percentage of facilities in each state that had required equipment and supplies for insertion and removal of contraceptive implants. Last, we used provider survey data to compute the number and percentage of health providers who reported having had challenging and difficult removals. Descriptive statistics were used to summarize the data Including mean, range and standard deviation.

The study investigator (AD) transcribed all qualitative interviews; transcriptions were validated by JE. The study investigator (AD) and the data manager/statistician (JE) independently reviewed all qualitative transcripts to get an overall sense of the data and to identify emergent themes in the data; AO reviewed transcripts to refine and clarify themes to develop a coding scheme. The coding scheme used predetermined categories and sub-categories derived from the four priority client-centered conditions and emergent themes. Framework analysis using Microsoft Excel was used to code transcripts and map and interpret findings. Illustrative quotations from the coding process were selected to add depth and context to the quantitative findings.

All respondents were deidentified during transcription (prior to analysis) to maximize confidentiality: each interview was assigned a unique ID linked to its specific catchment area. Personal identifiers were removed from notes and audio-recordings after transcription, and data were aggregated so no information could be traced to particular respondents. All recordings were deleted after transcription quality was confirmed.

### Ethical considerations

Prior to initiating the study, researchers first obtained permission for the study from federal and state ministries of health, implementing partners, the Hospital Management Board, the State Primary Health Care Agency, and hospital management. After being informed about the study objectives and procedures, and assured of confidentiality, all study subjects, facility managers and key stakeholders that participated in the study provided informed consent; no personally identifying information was collected.

A non-research determination approval was obtained from the Institutional Review Board at the Johns Hopkins Bloomberg School of Public Health (NRD #179). Ethical approvals were also obtained from the FMOH Research Ethics Committee of Nigeria (NHREC/01/01/2007-02/11/2018), and the State Ministries of Health in Ebonyi and Zamfara states before the assessment. Ethical approval was obtained from the Health, Research, and Ethics Committee in Nigeria at national and state levels.

## Results

Key characteristics of participating health facilities in both states are provided in Table 1 (the sample is detailed in Supplementary Table S1). The overwhelming majority of implant insertions and removals were provided on-site in facilities; in Zamfara, a small number of implant services were reported to have been provided through outreach. The study facilities in each state reported approximately the same number of insertions (approximately 270 per month); the ratio of insertions to removals was 8 to 1 in Ebonyi and 6 to 1 in Zamfara.

**Table 1:** Health facility characteristics related to implant insertions and removals in Ebonyi and Zamfara States, Nigeria.

Characteristic	Ebonyi State (*n* = 6)	Zamfara State (*n* = 6)	All Study Facilities (*N *= 12)
**Facility type, No. (%)**	** **	** **	**Total (%)**
Primary health center	2 (33.3)	2 (33.3)	4 (33.3)
Secondary hospital	2 (33.3)	2 (33.3)	4 (33.3)
Tertiary hospital	1 (16.7)	1 (16.7)	2 (16.7)
Private health facility	1 (16.7)	1 (16.7)	2 (16.7)
**Facility ownership, No. (%)**	** **	** **	**Total (%)**
Federal Ministry of Health	1 (16.7)	1 (16.7)	2 (16.7)
State or regional Ministry of Health	4 (66.6)	4 (66.6)	8 (66.6)
Private/Faith based	1 (16.7)	1 (16.7)	2 (16.7)
**Insertions per facility per month, mean (SD)**	** **	** **	**Mean**
Onsite	193.0	188.8	190.9
Outreach	0 (0.0)	3.5	1.75
**Removals per facility per month, mean (SD)**	** **	** **	**Mean**
Onsite	24.7	33.2	29.0
Outreach	0.0	3.5	1.8
**Percentage of implant removals per month, %**	11.3	16.5	**Percent** 13.9
**Providers per facility offering implant services, mean (SD)**	** **	** **	**Mean (SD)**
Insertions	1.1	2.1	2.4
Removals	1.1	2.1	2.4
Difficult removals	0	1.1	0.5

SD, standard deviation.

### Availability of competent and confident provider

All facilities assessed had at least two implant service providers, but across all levels of facilities, average numbers of providers per facility was higher in Zamfara than in Ebonyi. There was no difference in the average number of providers offering insertion and removal services. Tertiary facilities in Zamfara State had more providers available than other facilities. The number of providers skilled in performing difficult removals was very low: Ebonyi had none, and in Zamfara, the few providers who could manage difficult removals were concentrated in tertiary hospitals.

Most providers (90 percent) had adequate knowledge of key steps for removing contraceptive implants. Knowledge was slightly lower among providers in Ebonyi State (80 percent) compared to Zamfara State (100 percent). Eighty percent of providers expressed confidence in their ability to insert and remove implants; on average, provider confidence was higher in Zamfara than Ebonyi, and higher for insertion than removal (Table 2). Interestingly, while 90 percent of providers correctly identified the steps for removal, only 70 percent reported feeling confident in performing removals (Table 2). However, even where assessments measure adequate knowledge about removal, one respondent noted:

**Table 2:** Health provider characteristics related to implant insertions and removals in Ebonyi and Zamfara States, Nigeria.

Characteristic	Ebonyi State (*n* = 15)	Zamfara State (*n* = 15)	Total (*N* = 30)
**Confidence and knowledge, No. (%)**			
Confident with implant insertions	9 (60.0)	15 (100.0)	24 (80.0)
Confident with implant removals	9 (60.0)	12 (80.0)	21 (70.0)
Correct knowledge on implant removals^a^	12 (80.0)	15 (100.0)	27 (90.0)
**Lack of clients for practice during training, No. (%)**	6 (40.0)	2 (13.3)	8 (26.6)
**Supportive supervisory visit received by health providers in the last 3 months**	**4** **(26.7)**	**3** **(50.0)**	7 (23.3)

SD,  standard deviation.

^a^ Providers were asked to order a sequence of steps for one-rod implant removal.

*The situation in reality could be different when [providers] are observed unnoticed*. (Implementing partner, Ebonyi state)

Discussions with providers and stakeholders about improving provider knowledge and competency primarily revolved around training. Although training materials were designed based on industry standards, and implementing partners reported being involved in development of training manuals for different cadres of providers authorized to provide implant services, adherence to training standards varied. Key informants shared that while trainings were conducted using the national manual, duration and content varied. The revised pre-service curriculum, adopted in 2016, is general and does not outline key steps for implant insertion and removal. Particularly for difficult implant removals, the current LARC training manual may not suffice:

*The current LARC training manual does not adequately address [difficult removals] in line with recognized standards and no formal trainings of FP providers on difficult implant removal have been conducted in-country.* (FMOH Directorate respondent, National level)

Another implementing partner from the FMOH Directorate reported that FMOH had planned to organize training to build the capacity of FP providers to conduct difficult removals, but the initiative had never been implemented. One respondent linked lack of appropriate training with perceptions that removals were difficult:

*In one of our focal states, we received reports of difficulties with removal of implants [technical difficulties during the process of removal] from providers. Our investigations revealed it was due to activities of community health extension workers who had received on-the-job training from their colleagues.* (FMOH Directorate respondent, National level)

Several KII respondents reported that getting enough clients for removals presents difficulties in developing and maintaining skills:

*Getting removal cases during training can be challenging, we had to make do with models which is not very suitable for removal practice.* (Implementing partner, Ebonyi state)

*One major challenge is getting enough clients for removal practice, though not surprising as expected massive [numbers of] removals are still awaited.* (Implementing partner, National level)

Difficult removal cases were often avoided during training with real clients, limiting training opportunities to practice difficult removal:

*Typically, difficult cases of removals are excluded for live practice during training.* (Implementing partner, Zamfara state)

Even if a provider had received training on implant removal, they reported limited opportunities during and after training to develop and hone implant removal skills. For example, 26.6 percent of providers reported experiencing a lack of clients during training (Table 2). This challenge was more prevalent in Ebonyi State (40 percent) than in Zamfara (13.3 percent), where providers, especially those from the tertiary hospital where LARCs were more recently introduced, reported a lack of clients for practice. In these settings, most providers are trained using anatomical models:

*Due to inadequate number of clients for removals, we often have to complement with practice on anatomical models, which is much easier for insertion practice than for removals*. (Implementing partner, National level)

The United Nations Population Fund and implementing partner NGOs have furnished anatomical models to nursing and midwifery schools and pre-service institutions to support implant curricula, but KII respondents indicated that additional models are needed to facilitate training and accommodate growing numbers of students.

Of the 12 facilities, seven (58 percent) had job aids on family planning counseling and three (25 percent) had job aids on implant removal; only one facility (8 percent) had guidelines for nonpalpable implants. Supportive supervisory visits were infrequent: only 58.3 percent of facilities had received a visit from government authorities or implementing partners during the three months prior to the assessment (Table 3). KII respondents confirmed this lack:

**Table 3:** Client-centered conditions related to implant insertions and removals at health facilities in Ebonyi and Zamfara States, Nigeria.

Characteristic	Ebonyi State (*n* = 6) No. (%)	Zamfara State (*n* = 6) No. (%)	Total (*N* = 12) No. (%)
**Availability of all routine equipment and supplies**			
Insertion equipment and supplies	**0** **(0.0)**	**0** **(0.0)**	**0** **(0.0)**
Removal equipment and supplies	**0** **(0.0)**	**0** **(0.0)**	**0** **(0.0)**
Infection prevention supplies	**3** **(50.0)**	**1** **(16.7)**	**4 (25.0)**
**Availability of key equipment/instruments for difficult removal**			
Equipment to support difficult removals^a^	**0** **(0.0)**	**0** **(0.0)**	**0** **(0.0)**
Technology to support difficult removals^b^	**1** **(16.7)**	**2** **(33.4)**	**3** **(25.0)**
**Systems for managing difficult implant removals**			
Capacity for difficult or nonpalpable removals	**2** **(33.3)**	**2** **(33.3)**	**4** **(33.3)**
Mentorship program	**0** **(0.0)**	**0** **(0.0)**	**0** **(0.0)**
Quality improvement team	**0** **(0.0)**	**0** **(0.0)**	**0** **(0.0)**
Job aids available for FP counseling^c^	4 (66.7)	**3** **(50.0)**	7 (58.3)
Job aids available for implant removal	**1** **(16.7)**	**2** **(33.3)**	**3** **(25.0)**
**Data collection, review, and use**			
Both insertions and removals recorded in official register	6 (100.0)	4 (66.7)	10 (83.3)
Indications of implant removal recorded in official register	4 (66.7)	**2** **(33.3)**	**6** **(50.0)**
Data summary displayed^d^	**2** **(33.3)**	**0** **(0.0)**	**2** **(16.7)**
Data review meetings	**0** **(0.0)**	**0** **(0.0)**	**0** **(0.0)**

**Bold text** indicates that 50% or fewer facilities reported having or doing the item.

^a^ x-ray, ultrasound, and no-scalpel vasectomy forceps.

^b^ x-ray, ultrasound.

^c^ Family planning service delivery, including implant insertion and removal.

^d^ Facility displays data on family planning services (e.g., on a wall chart).

*We have not received any supervisory visits in the last three months.* (Health provider, Secondary health care facility, Ebonyi state)

*Supportive supervision is irregular and mostly conducted by non-governmental organizations*. (Health provider, Primary health care facility, Zamfara state)

Despite a provision for a mentorship program in Nigeria's FP blueprint, no structured mentorship programs for removals or quality improvement committees were in place at any facility.

### Supplies and equipment in place

Providers offering implant removal services reported limited access to adequate supplies and equipment. No facilities had all of the instruments required for implant insertions and removals despite actively providing the procedures (Table 3). Only one-quarter of facilities had all required infection prevention materials. Facilities lacked comprehensive equipment and instruments required to facilitate difficult removals; for example, modified vasectomy forceps, which are highly recommended for difficult removals, were not available at any facility (Table 4). In KIIs, only one RH coordinator reported that their state organizes bi-monthly review meetings to reconcile data on family planning commodities and provision for new stocks.

**Table 4:** Availability of equipment and supplies needed for insertion and removal of contraceptive implants by type of facility in Ebonyi and Zamfara States, Nigeria.

Item	Ebonyi and Zamfara States				Total (*N *= 12) No. (%)
	Tertiary hospital (*n* = 2) No. (%)	Secondary hospital (*n* = 4) No. (%)	Primary health center (*n* = 4) No. (%)	Private health facility (*n* = 2) No. (%)	
**Equipment and supplies for routine implant insertions and removals**			
Kidney dishes	2 (100.0)	4 (100.0)	4 (100.0)	2 (100.0)	12 (100.0)
Gallipot	2 (100.0)	4 (100.0)	4 (100.0)	2 (100.0)	12 (100.0)
Mosquito artery forceps (straight)	2 (100.0)	3 (75.0)	4 (100.0)	2 (100.0)	11 (91.7)
Mosquito artery forceps (curved)	**1** **(50.0)**	**2** **(50.0)**	3 (75.0)	2 (100.0)	8 (66.7)
Surgical blade	2 (100.0)	4 (100.0)	**2** **(50.0)**	2 (100.0)	10 (83.3)
Surgical handle	**1** **(50.0)**	3 (75.0)	3 (75.0)	2 (100.0)	9 (75.0)
Green towel	**1** **(50.0)**	4 (100.0)	**1** **(25.0)**	2 (100.0)	8 (66.7)
Lidocaine without epinephrine	2 (100.0)	**2** **(50.0)**	3 (75.0)	2 (100.0)	9 (75.0)
Sterile gauze	2 (100.0)	**2** **(50.0)**	**1** **(25.0)**	2 (100.0)	7 (58.3)
Sterile band aid or Elastoplast®	**0** **(0.0)**	**2** **(50.0)**	**2** **(50.0)**	2 (100.0)	**6** **(50.0)**
Sterile gloves	**0** **(0.0)**	3 (75.0)	3 (75.0)	2 (100.0)	8 (66.7)
Trocar	2 (100.0)	3 (75.0)	4 (100.0)	2 (100.0)	11 (91.7)
Povidone iodine	2 (100.0)	3 (75.0)	**2** **(50.0)**	2 (100.0)	9 (75.0)
Needle	2 (100.0)	**2** **(50.0)**	**1** **(25.0)**	2 (100.0)	7 (58.3)
Syringe	2 (100.0)	**2** **(50.0)**	**1** **(25.0)**	2 (100.0)	7 (58.3)
**Equipment and supplies for difficult implant insertions and removals**					
Modified vasectomy straight (blunt) forceps	**0** **(0.0)**	**0** **(0.0)**	**0** **(0.0)**	**0** **(0.0)**	**0** **(0.0)**
Ultrasound (5 MHz or 1O MHz)	2 (100.0)	**2** **(50.0)**	**0** **(0.0)**	2 (100.0)	**6** **(50.0)**
x-ray Machine	2 (100.0)	**1** **(25.0)**	**0** **(0.0)**	2 (100.0)	**5** **(41.7)**
**Other equipment**					
Autoclave in working condition	2 (100.0)	**1** **(25.0)**	**1** **(25.0)**	**1** **(50.0)**	**5** **(41.7)**
Examination couches	2 (100.0)	**2** **(50.0)**	3 (75.0)	2 (100.0)	9 (75.0)
Source of light	**1** **(50.0)**	**2** **(50.0)**	**2** **(50.0)**	**1** **(50.0)**	**6** **(50.0)**
Epinephrine	**1** **(50.0)**	**1** **(25.0)**	**0** **(0.0)**	2 (100.0)	**4** **(33.3)**
IV fluids	**0** **(0.0)**	**0** **(0.0)**	**0** **(0.0)**	**0** **(0.0)**	**0** **(0.0)**
**Infection prevention supplies**					
Running water	**1** **(50.0)**	**2** **(50.0)**	**2** **(50.0)**	**1** **(50.0)**	**6** **(50.0)**
Decontamination buckets	1 (100.0)	4 (100.0)	4 (100.0)	2 (100.0)	12 (100.0)
Safety boxes	1 (100.0)	3 (75.0)	4 (100.0)	2 (100.0)	11 (91.7)
Soap	2 (100.0)	3 (75.0)	**2** **(50.0)**	2 (100.0)	9 (75.0)
Chlorine	2 (100.0)	4 (100.0)	**2** **(50.0)**	**1** **(50.0)**	9 (75.0)

**Bold text** indicates items that 50% or fewer facilities reported having.

Although ultrasound and x-ray machines were available at both tertiary health facilities, none of the primary health facilities had these machines (Table 4). Five providers (16.6 percent) reported challenges with equipment or instruments, primarily inadequacy of instruments (Table 5). Overall, 53.3 percent (16 of 30) of providers had encountered clients unable to have their implants removed when desired, usually due to lack of equipment or supplies (Table 5).

**Table 5:** Health provider reports of challenging and difficult removals in Ebonyi and Zamfara States, Nigeria.

Characteristic	Ebonyi State (*n* = 15)	Zamfara State (*n* = 15)	Total (*N* = 30)
**Clients with difficult removals, No. (%)**					
0	10 (66.7)	8 (53.3)	18 (60.0)
1–2	5 (33.3)	7 (46.7)	12 (40.0)
≥3	—	—	0 (0.0)
**Challenges removing an implant, No. (%)** **^a^**					
Any challenge	7 (46.7)	14 (93.3)	21 (70.0)
Case-specific challenges			
Deeply inserted implant	7 (46.7)	11 (73.3)	18 (60.0)
Excessive bleeding	2 (13.3)	1 (6.7)	3 (10.0)
Equipment and supply challenges			
Lack of equipment or instruments	0 (0.0)	1 (6.7)	1 (3.3)
Any challenge with equipment function	2 (13.3)	3 (20.0)	5 (16.7)
Power failure	0 (0.0)	1 (33.3)	1 (20.0)
Rusting of instrument	0 (0.0)	1 (33.3)	1 (20.0)
Not enough instruments	2 (100.0)	1 (33.3)	3 (60.0)
**Reason clients failed to have an implant removed, No. (%)**					
Any	7 (46.7)	9 (60.0)	16 (53.3)
Provider attempted, but could not remove	2 (13.3)	0 (0.0)	2 (6.7)
Equipment or supplies not available	6 (40.0)	1 (6.7)	7 (43.8)
Equipment not processed	0 (0.0)	1 (6.7)	1 (6.3)
Cost too high	0 (0.0)	1 (6.7)	1 (6.3)
Provider too busy	0 (0.0)	6 (40.0)	6 (20.0)
**Course of action, No. (%)**					
Refer to colleague	10 (66.7)	4 (26.6)	14 (46.6)
Refer to doctor at the same hospital	5 (33.3)	7 (46.6)	12 (40.0)
Refer to another hospital	0 (0.0)	3 (20.0)	3 (10.0)
Remove implant	0 (0.0)	1 (6.6)	1 (3.3)

SD, standard deviation.

^a^ Providers were asked about six case-specific challenges and six challenges related to equipment or supplies (Appendix C).

### System in place for difficult implant removals

Systems for handling difficult removals were inadequate. Only four facilities (33.3 percent) had capacity to support removals of difficult or non-palpable implants. Seventy percent of providers reported having encountered a difficult removal, usually due to deep insertion (reported by 60 percent of providers), though this was reported much more commonly in Zamfara (93.3%) than Ebonyi (46.7 percent). Only two providers, both from Ebonyi, reported a failed attempt at removal. Only one health facility (in Ebonyi State) had a guideline for managing non-palpable implants.

When faced with difficult removals, providers most commonly referred clients to a colleague (46.6 percent) or a doctor at the same facility (40.0 percent), usually with more experience:

*Anytime I have difficulties with implant removal I call on my colleague who is more experienced.* (Midwife, secondary health care facility, Zamfara state).

Most providers at tertiary hospitals referred difficult implant removal cases to doctors (80 percent of providers in Ebonyi and 75 percent in Zamfara, respectively). The majority of Ebonyi providers referred difficult removals to colleagues (66.7 percent), particularly providers at primary (and secondary facilities (100 and 80 percent, respectively). Most commonly, providers assessed in Zamfara (46.6 percent) referred difficult removal clients to doctors (Table 5). Only 10 percent of providers—all in Zamfara—referred patients to another hospital.

Providers corroborated the general lack of systems to manage difficult removals:

*We don't have any written guideline for referral of difficult to remove implants but we often refer to doctors within the hospital.* (Facility head, tertiary hospital, Ebonyi state)

*We had a particular client referred to us whose implant we could not remove, and we had to invite our trainers from headquarters during a rescheduled appointment. Unfortunately, they were also unsuccessful, and the client had to be referred to the teaching hospital for further action. It was very distressing as the husband of this particular client was not aware that she is using any form of contraception*. (Provider, primary health facility, Ebonyi state)

*Our facility does not have a quality improvement team or mentorship program in place, although we have a consultant [gynecologist] who gives us support when we have difficulties with implant removals*. (Facility head, tertiary hospital, Zamfara state)

### Implant removal data collected and monitored

Removal procedures were captured by half of the facilities, though data collection was not standardized. Most health facilities (83.3 percent) used the national FP register to capture the number of clients receiving implant insertion and removal services, including all facilities in Ebonyi state and three of six facilities in Zamfara (Table 3). Indications were most often documented in the remarks column of the FP register. One facility in Zamfara State maintained a separate register and an exercise book to capture implant removals:

*In our facility, we document indication for removals in exercise books, because there is no provision in the register.* (Facility head, tertiary hospital, Zamfara state)

Difficult removals were not recorded by any of the facilities reviewed, though some documented indications in the remarks column:

*The register we use does not have a column to capture indications for removal or difficulties with removals.* (Facility head, primary health care facility, Ebonyi state)

Only two facilities displayed monthly data summaries; no facilities organized monthly review meetings (Table 3).

Implementing partners reported participating actively in the National Reproductive Health Technical Working Group during quarterly reproductive health meetings. Some partners use digital apps to track FP service delivery (examples include the Health Network Quality Improvement System and CommCare), but none specifically include implant removals. One implementing partner operates a contact call center to support client follow-up and tracking, and provides feedback on services received at facilities. However, none of these tracking or feedback mechanisms is specifically deployed for monitoring implant removals. A KII respondent at FMOH noted that the family planning dashboard captures insertions but not removals:

*At country level, we have a family planning dashboard that captures implant insertions; however, the dashboard does not capture implant removals.* (FMOH Directorate respondent, National level)

## Discussion

Sustained support for quality implant removals will be required to keep pace with insertions and meet client-centered conditions for quality service in Nigeria. The results of the landscape assessment suggest that to achieve these goals, specific investments are needed in training, structured mentorship programs, and supportive supervision (especially in primary facilities); facility readiness to perform implant removals; systems to manage difficult removals; and collection and use of data on implant removals.

### Strengthen provider competency and confidence in implant removal

Although the number of providers performing implant insertions has been rapidly scaled up, shortages of confident and competent providers are still present, particularly outside of tertiary hospitals. Non-standard training content, lack of structured mentorship programs, and low coverage of supportive supervision for providers in implant removal suggests areas where investments are needed to ensure providers can perform quality implant removal, especially in cases of difficult implant removal and where task-shifting strategies are adopted. Training opportunities that foster clinical competency are needed for training in removals: Christofield et al. reported that a substantial percentage of providers in Uganda and Kenya never practiced implant removal on a client during training (18). In Senegal, Lebetkin et al. reported substantially higher percentages of providers who felt confident in performing uncomplicated removals (96 percent) than in our Nigeria assessment, but 15 percent did not feel confident removing non-palpable implants and an additional 15 percent did not provide this service (36).

Importantly, no formal trainings on difficult implant removal have been conducted in Nigeria. Competency-based training both for implant removal and difficult removal can support both service quality and provider confidence: conversely, nurses in South Africa blamed brief cascade trainings with inadequate content for their lack of confidence in providing implant removal (37). In addition to ensuring that pre-service and in-service trainings cover difficult removal, existing LARC curricula—most centrally, Nigeria's national manual for physicians and nurse-midwives—must be reviewed for completeness, and supportive supervision and mentorship programs should be used to support provider skills, particularly as task-shifting necessitates training and support for new cadres (10, 38, 39). There was a striking difference between providers in Ebonyi and Zamfara states having encountered difficult removals, possibly because in Zamfara, implant services can be provided by community health extension workers who may be less equipped to manage removals generally, particularly removals they perceive as challenging. As Nigeria increasingly relies on trained community health extension workers for expansion of provision of implant insertion and removal services, there is a risk of delayed removal resulting from greater numbers of insertions at the community level without access to quality removal services—as was observed in Ethiopia after a similar strategy was adopted (31). As it may not be feasible to establish quality removal services at all levels of the health system, this risk should be addressed through concurrent efforts to establish and strengthen referral networks for difficult removals, like those piloted in northern Nigeria by Charyeva et al. (10). Toolkits, job aids, modeling exercises, and curricula to train providers in LMICs in standard and difficult implant removal already exist that could be adapted for settings where implant use is rising, including management of side effects and managing difficult removals (40–42).

### Ensure adequate supplies and equipment for implant removal

No surveyed facilities had adequate materials for implant service delivery. A lack of required infection prevention materials in three-quarters of the survey facilities, as well as infrastructural deficiencies like lack of running water, compromise quality of care and increase the risk of adverse client outcomes. Supply stockouts are common challenges in providing implant removal services: a study from Uganda found that only 8 percent of facilities providing implant removal services had the necessary instruments and consumables for the procedure, and in Ethiopia, service providers identified stockouts of appropriate supplies as the reason for their inability to provide removal services (18, 43). Of particular concern in this Nigerian study context is the scarcity of specialized equipment needed to manage difficult removals, including ultrasound and x-ray, and the fact that no facilities had modified vasectomy forceps. Similar deficiencies have been noted in studies in other sub-Saharan African countries: only 8 percent of facilities assessed in Senegal were fully equipped to manage difficult removals (23). Stockouts also have adverse impact on providers' ability to perform quality removals, but despite national commitment to offer free FP services, a lack of formal policy and regulation relating to the private sector stymies effective public-private partnerships needed to sustain robust supply chains (44).

### Develop systems to manage difficult implant removals

Systems to manage difficult implant removals were scarce and insufficient. Providers themselves conflated clinical indications (e.g., migrated or deeply inserted implants) with other technical challenges and difficulties with removal (e.g., lack of adequate equipment or skill to remove an implant), suggesting the need to develop and adopt a technical definition for difficult removals, but also to recognize that providers often encounter obstacles even when performing standard removals. Howett et al. describe an essentially identical context in Botswana, finding that nine of ten providers reported having experienced barriers to providing implant removal, including inadequate training, insufficient equipment, insufficient time, and lack of a referral pathway for difficult removals (45). Organized referral networks may offer a means for clients with difficult removals to obtain quality services in health systems with limited capacity: in Senegal, 72 percent of facilities in organized referral networks referred clients with difficult removals to other facilities, much higher than in this Nigeria landscape assessment where referral out was uncommon (23).

Building capacity to recognize, manage, and refer difficult removals to facilities and providers that can provide quality removal is essential, especially at primary and secondary health facilities. Difficult removal must be included in training curricula, alongside efforts to improve provider skill in performing difficult removals and strengthening referral systems for difficult removals. Increasing availability of ultrasound is another important strategy in developing systems for difficult removals: Petro et al. observed that 92 percent of referred cases reaching their referral facility in South Africa were for nonpalpable implants, 97 percent of which were removed successfully with ultrasound localization in an outpatient setting (46). Supporting the development of model centers for implant services and referral sites—including outpatient clinics—for difficult removals with ultrasound equipment and appropriate instruments could be effective strategies to improve access to implant removal services.

### Systematically collect and use data on implant removals

To track the performance of efforts to expand implant use, there is a need for routinized collection of data on implant removals, as well as reasons for method switching or discontinuation. It is encouraging that most facilities (and the DHIS-2 and FP dashboard) tracked insertions, but numbers of removals were undercounted and indications for difficult removal are unknown. These are both essential indicators for expanding and evaluating implant services. As in Nigeria, LARC removals are not routinely captured in health facility registers or the national HMIS. In Mozambique, Jacinto et al. note that documenting outcomes of LARC removals could help health management teams identify increases in difficult removals and encourage assessment of provider competencies and adequacy of supplies and equipment (21). Governments should consider including removal indicators in their national HMIS to strengthen monitoring of family planning initiatives to improve the quality of care. We found no evidence that facilities actively use data to support quality assurance or quality improvement. An ongoing review of HMIS data collection and reporting forms, initiated in November 2018, may provide an opportunity to track and include implant removal indicators in Nigeria's HMIS tool. Advocating for the inclusion of implant removal indicators on facility registers and in national dashboards could powerfully advance evidence-based decision-making around contraceptive implant programs. In conjunction with improving the quality of implant insertion and removal data, program and policymakers must also learn how to interpret removal data, including recognizing indications of underreporting, barriers to removal services, and higher than expected discontinuation rates.

### Strengths and limitations

Including multiple data sources in the assessment facilitated wide stakeholder engagement and allowed triangulation of findings. Some interviews were completed either virtually or in-person during multi-stakeholder conferences for convenience; while some virtual interviews experienced technical difficulties such as dropped calls, there was no noticeable difference in rapport or quality of data collected. Still, small sample sizes, limited by logistic and funding constraints, may adversely affect the generalizability of the findings. The landscape assessment included just two of Nigeria's 36 states, and six facilities per state. Additionally, only facilities already offering implant removal services as a part of routine service delivery were considered for inclusion.

While the assessment measured provider knowledge around implant services, clinical service delivery was not monitored; provider knowledge may not correlate with actual practice. Additionally, provider confidence was self-assessed and subjective, and may have been overestimated due to social desirability bias. We did not collect information about providers' clinical work experience or how long they had been providing implant removal services, and did not disaggregate the analysis by type of provider (though generally, primary level care is provided by community health workers, secondary level by nurse-midwives, and tertiary level by doctors and nurse-midwives). We are thus unable to explore how confidence and knowledge may vary by type of provider and experience in implant removal. Similarly, because difficult removals were not captured in facility registers, the assessment relied on provider recall of difficult removals based on how providers subjectively defined “difficult removal,” which was much broader than clinical indications for difficult removal. This definitional variation and recall bias may have hindered providers' ability to accurately estimate the burden of difficult removals.

### A roadmap for quality implant removal

The decade ahead is one of promise: Nigeria has committed to scaling up rights-based, high-impact practices for family planning that meet the needs of individuals and families and to increasing modern contraceptive prevalence to at least 27 percent by 2030 (47). To achieve this milestone by 2030 and ensure FP choice for future generations requires collective action, particularly to make good on the promise made to contraceptive implant users upon insertion that their method can be discontinued when desired. Actions taken at multiple levels—to improve provider and facility readiness, supply sites with adequate equipment, and utilize data effectively to monitor and address potential issues—could yield incredible progress. With this vision in mind, the landscape assessment results were shared nationally through a dissemination and co-design workshop to develop a roadmap for key actions in partnership with the FMOH and FP implementing partners (Table 6).

**Table 6:** Roadmap to providing quality implant removal services.

Client-centered condition for quality implant removals			
Strengthen provider competency and confidence in implant removal	Ensure adequate supplies and equipment for implant removal	Develop systems to manage difficult implant removals	Systematically collect and use data on implant removals
• Include difficult implant removal as gynecology and surgery competency, including US localization and removal of nonpalpable implants • Build tutor and preceptor capacity to teach implant removal, including offering clinical training skills standardization trainings • Update and standardize LARC training curricula for pre-service and in-service settings to include implant removals, including difficult removals • Incorporate US localization and removal of nonpalpable implants as compulsory competencies in in-service trainings • Select high-volume facilities for training so live clients are available • Develop compliance protocols to improve adherence to content and duration of trainings • Advocate for quarterly supportive supervisory visits from MOH to LARC-providing facilities • Foster mentorship of inexperienced health workers by highly skilled providers of implant removals in their facilities or nearby	• Inventory health facilities and advocate in local government areas for needed equipment and consumables (especially infection control) for insertions, removals, and difficult removals • Include modified vasectomy forceps in standard supply inventories • Develop, print, and disseminate job aids on removal to all LARC providers (including community health extension workers), and video job aids for referral centers • Ensure pre-service trainers have adequate anatomical models for implant removal	• Include FP unit rotations in medical residencies • Include surgeons in non-palpable localization and removal skill-building activities • Identify difficult removal master trainers • Map providers and facilities capable of performing difficult removals • Develop referral protocol for difficult removals • Develop model centers for referral and sensitize frontline providers on referral there in cases of difficult removal	• Advocate to ensure that HMIS tools collect critical data on numbers of and indications for implant removal for inclusion in the DHIS2 and the FP dashboard • Encourage proper recordkeeping and documentation of implant removals at facilities • Support data review meetings at facility, district, and state levels • Identify platforms for provider communication and peer feedback on challenging implant removals (e.g., *via* WhatsApp); monitor persistent challenges

Expanding capacity to provide implant removals by improving facility readiness to provide implant removals at primary and secondary health facilities will require revising training curricula to include a module on difficult implant removals, developing appropriate job aids, and formulating clear referral procedures. These investments will ensure that quality removals are available when women require them. Bolstering procurement systems and supply chains to ensure availability of implant removal supplies and equipment—and a free, accessible contraceptive method of choice for every client—will require good clinical governance at the facility level, productive partnerships with the private sector, and political commitment and engagement at the local and state levels.

Data management systems require investment to improve coordination, analysis, and utilization of data on implant services for evidence-based decision making at the facility, local, state, and national levels. Documentation and review of indications for and numbers of implant removal procedures are critical inputs for evidence-based decision-making. Facilities need support to routinely collect data about implant insertions and removals in registers, including reasons for removal; adding implant removal indicators to the HMIS and FP dashboards will permit continuous monitoring of the quality of implant services.

## Conclusion

The need for implant removals will grow in tandem with increasing implant uptake in Nigeria and across sub-Saharan Africa. Roadmaps like that offered for Nigeria through the Expanding Family Planning Choices Program are needed to ensure routine, regular, and reliable removal services for clients, including strategic planning to support implant removals alongside service expansion efforts. Improved quality of service supports continued uptake of implants and contraceptives more generally, key to the success of FP programs everywhere.

## Data Availability

The data presented in the study are deposited in the FigShare repository, accession number https://doi.org/10.6084/m9.figshare.22259614.v1.
