# Supplementary material for: What is needed to improve quality of implant removal services in Nigeria? results of a landscape assessment
Source: Front Glob Womens Health. 2023 Mar 22;4:1082969. doi: 10.3389/fgwh.2023.1082969 (PMC10073656; doi:10.3389/fgwh.2023.1082969)
Supplement: Supplementary file 1 [file Table1.docx]

**Supplementary Table S1: Sample size**

| **Semi-structured survey of facility managers on facility readiness &**  **Service statistics from HMIS** | | | | | |
| --- | --- | --- | --- | --- | --- |
| State | Tertiary  health  facilities | Secondary health  facilities | Primary health centers | Private  hospitals | Total |
| Ebonyi | 1 | 2 | 2 | 1 | 6 |
| Zamfara | 1 | 2 | 2 | 1 | 6 |
| Total | 2 | 4 | 4 | 2 | 12 |
| **Semi-structured survey with providers on knowledge and confidence** | | | | | |
| State | Tertiary  health  facilities | Secondary health  facilities | Primary health centers | Private  hospitals | Total |
| Ebonyi | 5 | 5 | 4 | 1 | 15 |
| Zamfara | 4 | 5 | 4 | 2 | 15 |
| Total | 9 | 10 | 8 | 3 | 30 |
| **Qualitative KIIs with stakeholders at state and national level** | | | | | |
| State/Level | RH coordinators (#) | Implementing  partners (#) | Government  stakeholders (#) | Pre-service  institutions (#) | Total |
| Ebonyi | 1 | 1 | -- | 2 | 4 |
| Zamfara | 1 | 1 | -- | 1 | 3 |
| National | -- | 14 | 3 | -- | 17 |
| Total | 2 | 16 | 3 | 3 | 24 |
